# Cardia-AI: Passive Cardiac Event Monitoring Using Smartwatch Sensors and Predictive Analysis via Large Language Models

**DOI:** 10.7759/cureus.95083

**Published:** 2025-10-21

**Authors:** Elyan Ali Momin, Hamid Mansoor

**Affiliations:** 1 Computer Science, University of Manitoba, Winnipeg, CAN

**Keywords:** digital health, generative ai, large language models (llms), sensor data, wearable devices

## Abstract

Cardiovascular diseases require continuous, context-aware monitoring, i.e., combining day-to-day wearable signals with recent diagnoses, medications, and symptom reports, rather than isolated clinic visits or single-spot measurements. We developed Cardia-AI, a proof-of-concept pipeline that time-aligns smartwatch signals (heart rate, blood pressure, oxygen saturation) with a patient’s longitudinal electronic health record (EHR) and uses a compact medical language model with retrieval to produce grounded educational summaries. Cardia-AI assembles time-aligned summaries through a select, compile, and ask pipeline, and uses a lightweight medical large language model (BioMistral-7B) with retrieval from curated sources. Guardrails constrain outputs to education and navigation, and the system incorporates explicit escalation guidance when red-flag symptoms are present in the prompt context. In two scenario-based validations (cardiometabolic education; early post-angioplasty recovery), Cardia-AI compiled synchronized smartwatch trends with EHR entries, referenced the exact measurements and diagnoses present in the prompt, and recorded transcripts for audit and reproducibility; no outcomes or accuracy endpoints were assessed. We did not evaluate clinical effectiveness or diagnostic accuracy; no patient outcomes were measured. This work reports feasibility and safety guardrails only, with prospective evaluations planned. These demonstrations suggest that pairing wearable streams with a compact, domain-tuned language model may lower cognitive load from multi-panel charts and shorten the path from symptom onset to appropriate follow-up under clinician oversight.

## Introduction

Cardiovascular diseases (CVDs) remain the world’s leading cause of death and disability. The World Health Organization estimates that 19.8 million people died from CVDs in 2022, roughly one third of all global deaths, with the burden falling disproportionately on low- and middle-income countries and a substantial share occurring prematurely (before age 70) [1,2]. These realities underscore the need for earlier detection and tighter longitudinal management outside clinic walls, where much day-to-day deterioration actually unfolds [2].

Consumer wearables and medical-grade patches have transformed out-of-clinic cardiac observation. Photoplethysmography (PPG) on smartwatches, single-lead ECG sensors, adhesive patches (e.g., ZioPatch), and flexible textile sensors enable continuous tracking of rhythm, rate, oxygen saturation, and related metrics [3-5]. At the population scale, the Apple Heart Study demonstrated that an irregular-pulse notification algorithm could identify atrial fibrillation (AF) and coordinate confirmatory patch monitoring via a fully remote, “siteless” design - an important feasibility milestone for large pragmatic digital trials [3]. Meta-analyses report pooled sensitivities and specificities in the mid-90% range for AF detection with both smartwatches and chest patches, while noting variability across devices, contexts, and study designs [5,6]. Accordingly, robust systems should incorporate signal-quality checks, demographic-aware calibration, and clear escalation pathways to medical-grade diagnostics and clinician oversight [7].

Large language models (LLMs) can serve as a translation layer that turns heterogeneous cardiac data into patient-facing guidance and clinician-ready summaries [8-11]. We describe Cardia-AI, a prototype LLM-assisted monitoring system that fuses smartwatch signals (heart rate, oxygen saturation, blood pressure) with historical electronic health record (EHR) context to flag early signs of cardiac deterioration and generate patient-facing explanations for patients and clinicians. Cardia-AI combines smartwatch sensor-derived digital biomarkers with a compact biomedical language model (BioMistral-7B [12]) to convert multiday trends into succinct self-care prompts and visit-preparation briefs.

Given the safety and regulatory limits of current clinical LLMs (e.g., Med-PaLM 2, AMIE [13]), Cardia-AI constrains open-ended or “free-form” generation by using a retrieval-templated method. In this design, every response is drawn from curated cardiovascular sources and follows a structured template, in contrast to free-form systems that may produce unsupported statements. In practice, the template constrains sections of the output (what the issue is, why it matters, when/how to contact care) and requires inline citation of the measurements and diagnoses used, whereas free-form systems may assert content without provenance. The retrieval approach is modeled after the Almanac framework [14], which emphasizes source-grounded outputs and clinician-verification routes.

This work contributes to the technical feasibility of LLM-mediated cardiac education and navigation, with reference to current unmet needs in digital health. These contributions are feasibility-oriented and illustrative. They do not imply clinical effectiveness:

Architecture: A passive monitoring pipeline that collects smartwatch signals via device application programming interface (APIs), enriches them with EHR context, and applies personalized anomaly detection.

Explanation layer: An LLM-mediated interface (BioMistral-7B with retrieval) that generates concise rationales, visit-prep notes, and self-management prompts for common cardiac scenarios.

Use cases: Illustrative pathways (education and post-surgical recovery) showing how integrating wearable data, verified EHR snippets, and conversational queries can shorten the time from symptom onset to appropriate action without bypassing clinician oversight. For example, long-term hypertensive patients could use the system to understand trends in blood pressure and medication adherence, while post-angioplasty patients may benefit from contextual explanations of short-term heart rate variability.

Evaluation and safety: Practical considerations for continuous cardiac monitoring with LLM support, emphasizing signal quality, demographic calibration, provenance, and clinician-in-the-loop safeguards aligned with recent deployments in messaging triage and patient comprehension [11].

## Technical report

Methodology

Data Sources and Construction

We combined two primary data streams to simulate the end-to-end behavior of the prototype monitoring system. No real-world testing with patients was conducted in this feasibility study; all evaluations were limited to simulated smartwatch streams and de-identified EHR records. First, EHR data were drawn from MIMIC-IV [15], a publicly available, de-identified critical care dataset that contains admissions, diagnoses, procedures, medication exposures, and selected physiologic measurements. Second, simulated smartwatch data were sourced via Android Health Connect (Google LLC, Mountain View, CA; version 2024.1.0), a digital platform that provides access to users’ connected device data, to represent time-stamped heart rate and oxygen saturation observations during everyday activity. The Health Connect data were simulated and collected using an in-lab Google Pixel 3 smartwatch (Google LLC, Mountain View, CA; Android Wear OS version 2.23) by the authors over several days, and applied to the personas in the two use cases (described later) for demonstrative purposes. Simulations were generated on a standard workstation (Windows 11 Pro (Microsoft Corp., Redmond, WA), Dell Precision 3660 (Dell Technologies, Round Rock, TX), Intel i7 CPU (Intel Corporation, Santa Clara, CA), 16 GB RAM). All code was implemented in Python 3 (Python Software Foundation, Wilmington, DE) using NumPy and Pandas (NumFOCUS, Austin, TX). No proprietary device software was accessed; device APIs were emulated through Health Connect schema documentation (Google LLC, Mountain View, CA). To keep the focus on patient-facing explanation and care navigation, we describe only the components essential to the user workflow and omit nonessential implementation details.

Because the Pixel Watch does not natively capture blood pressure, we synthesized blood-pressure time series data (corresponding with the simulated Health Connect data) using a physiologically constrained generative procedure with defined parameters: diurnal rhythm with mean systolic 130 ± 15 mmHg, diastolic 80 ± 10 mmHg; autocorrelation coefficient ≈0.65 across one-hour lags; Gaussian measurement noise σ = 5 mmHg. “Real-world setting” here is defined as one in which continuous wearable measurements (e.g., HR, SpO₂), verified EHR diagnoses, and patient-reported symptoms are all simultaneously available to the Cardia-AI pipeline at the point of query. These sources jointly approximate the real-world setting in which a patient’s longitudinal clinical history and recent vital signs are available at the point of question and answer within the prototype system. In this manuscript, “real-world setting” refers to deployment on commodity smartphones and wearables outside controlled laboratory conditions, integrated with de-identified EHR data streams. No patient-facing deployment was performed in this feasibility stage.

Data Preparation and Normalization

EHR tables from MIMIC-IV were filtered to a cardiometabolic focus, specifically hypertension, type 2 diabetes, obesity, and coronary artery disease. Records containing admissions, problem lists, and procedure histories linked to these conditions were extracted, coded, and aligned to support the construction of the scenario personas. Medication information was aligned to admission and discharge windows to support later interpretation of vital sign changes around therapy adjustments. The simulated Health Connect records were cleaned and standardized into a common time base using rolling windows and daily aggregates. For clarity, signal processing is summarized as standard smoothing/aggregation and outlier handling to preserve salient trends (HR, BP pairs, SpO₂ events) without overloading the reader with low-level parameters. All timestamps were converted to a single time zone and stored with millisecond precision to maintain the order of events.

Identity, Storage, and Schema

All records were written to Firebase Firestore (Google LLC, Mountain View, CA, https://firebase.google.com/) under a hierarchical schema organized by user, data domain, and date. Within each synthetic user document, we created subcollections for vitals, self-reported items, and verified clinical artifacts. Each artifact retained its source tag, MIMIC-IV or Health Connect, and its provenance metadata, including the table of origin or device type when available. To align with the real-world application behavior, user-submitted records were assumed to be self-reported at first, with a simulated workflow for verification by healthcare professionals. We have given names to the patients and also added some details regarding their hospital admissions and dates, for readability purposes. The actual data remains unchanged.

Self-Report Capture and Clinician Verification Workflow

To reflect real application use, the patient-entered information was input through a self-report form and written to Firestore with a verification status of pending. A simulated clinician review process then reconciled these entries against the EHR view, promoting items to verified and, where appropriate, attaching clarifying notes. All transitions between pending and verified states were logged with timestamps and reviewer identifiers to produce an auditable trail. When verified items superseded or corrected self-reports, both the original and the corrected entries were preserved to maintain traceability.

Prompt Assembly and LLM Orchestration

**Figure 1:**
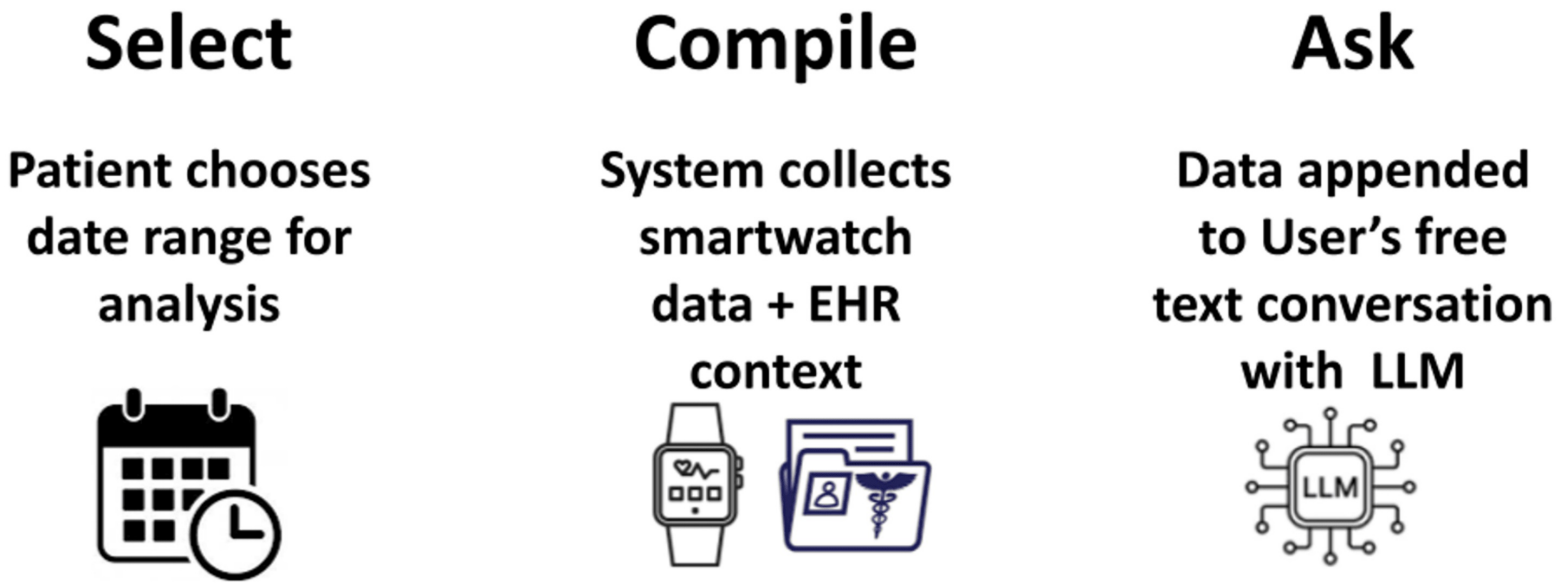
Select-Compile-Ask pipeline for Cardia-AI

The application generated question context through a select, compile, and ask pipeline (Figure 1). The user first selects a date range for Health Connect vitals. The application then compiles aggregated vitals, recent diagnoses, and procedures from Firestore for that window, highlighting salient patterns such as persistent morning hypertension or activity-linked heart rate surges. The resulting structured context was appended to the user’s free-text question and forwarded to the language model through the backend. Prior to dispatch, the text is scanned for pre-specified escalation criteria (e.g., chest pain, sustained tachycardia, or new syncope) terms and symptom combinations that require prominent escalation messaging in the eventual response.

Guardrails, Safety Messaging, and Escalation Thresholds

The language model was restricted to education and navigation only, i.e., it may summarize trends, explain plausible mechanisms, and advise when/how to contact care, and it does not render diagnoses, triage decisions, or medication advice. Responses were programmed so as to be required to cite which data elements informed the explanation by referencing time ranges and artifact names from the compiled context. The system had explicit escalation guidance when risk features were present. For example, persistent chest pressure with shortness of breath, dizziness, or nausea, and displayed options for contacting emergency services or the clinical team. Every interaction was recorded as a signed, versioned transcript that included the exact prompt, the returned answer, and the data snapshot used to create it.

Interaction Flow and User Experience

The user experience is centered on two actions: inspect and ask. Inspect rendered dashboards with rolling distributions and trends for heart rate, blood pressure, and oxygen saturation, overlaid with verified clinical events. Ask allowed the user to select a data window, preview the compiled context, add questions, and submit to the model. Answers were returned with plain language explanations, linked back to the underlying measurements and problem list items, and paired with concrete next steps such as gentle activity trials, symptom logging, or follow-up scheduling.

Evaluation and Reproducibility Practices

Since our aim was to demonstrate application behavior rather than to estimate clinical effectiveness, we used scenario-based validation anchored in the two demonstrative personas. For each scenario, we confirmed that the compiler included the correct data windows, that responses referenced the intended measurements and clinical entries, and that pre-specified escalation criteria logic displayed the appropriate escalation prompts. We did not seek outcome or accuracy estimates; progress is presented as the feasibility of the explanation workflow and safety checks.

Demonstrative use cases

This prototype supports patient-facing explanations and care navigation support and care navigation under clinician oversight and is not a diagnostic or treatment tool. Our application integrates three data sources, smartwatch vitals (from Android Health Connect), patient-entered self-reports, and clinician-verified EHR artifacts, to power an in-app, health-focused LLM that converts complex clinical information into plain language next-step guidance while preserving traceability to sources. The end-to-end interaction follows a structured sequence of select, compile, and ask. A patient chooses a time window for Health Connect data.

The system automatically aggregates vitals such as heart rate, blood pressure, and SpO₂, merges problem lists, procedures, and medications from the EHR, and constructs a context-rich prompt. The patient then appends free-text questions before submission. By anchoring model inputs to timestamped vitals and named EHR entries, the system minimizes copy and paste errors, reduces cognitive load from multi-panel charts, and makes explanations verifiable, which is essential for clinical audit and research reproducibility. To promote safety, the LLM is restricted by guardrails.

The prototype provides structured patient-facing explanations rather than diagnostic guidance, applies pre-specified escalation criteria (e.g., persistent chest pressure with shortness of breath, dizziness, or new syncope), and surfaces emergency service directives inline when such criteria are detected. Explanations are generated in plain, patient-friendly language, while remaining traceable by citing the specific measurements and problem list items that informed the answer. The project demonstrates workflow and user interface behavior and does not claim diagnostic or therapeutic capability. The cardiometabolic examples that follow are illustrative of how multi-source data can support directed education and preparation for clinician follow-up; we do not evaluate clinical outcomes or effectiveness in this study.

Privacy, Security, and Ethics

MIMIC-IV data are de-identified at the source. The Android Health Connect data in our demonstrations were simulated with an in-lab smartwatch, and the associated blood pressure data were synthetically created. Firestore access required authenticated service accounts with least privilege rules, and all collections enforced server-side validation of schema and provenance tags. No attempt was made to re-identify any subject, and no decisions intended for clinical care were generated.

These use cases are scenario-based demonstrations, not clinical trials, and were constructed using simulated smartwatch data and de-identified MIMIC-IV records to illustrate feasibility. We provide two use cases for the following personas that we collected from the de-identified MIMIC-IV database: a patient seeking longitudinal education across cardiometabolic conditions and a postoperative patient navigating early recovery. We have replaced the original dates from the MIMIC-IV database with placeholder dates in the figures below as an extra precaution. We have given the personas fictional names for readability:

Use Case 1: Patient Understanding of Health Trends via LLM-Based Navigation

**Figure 2:**
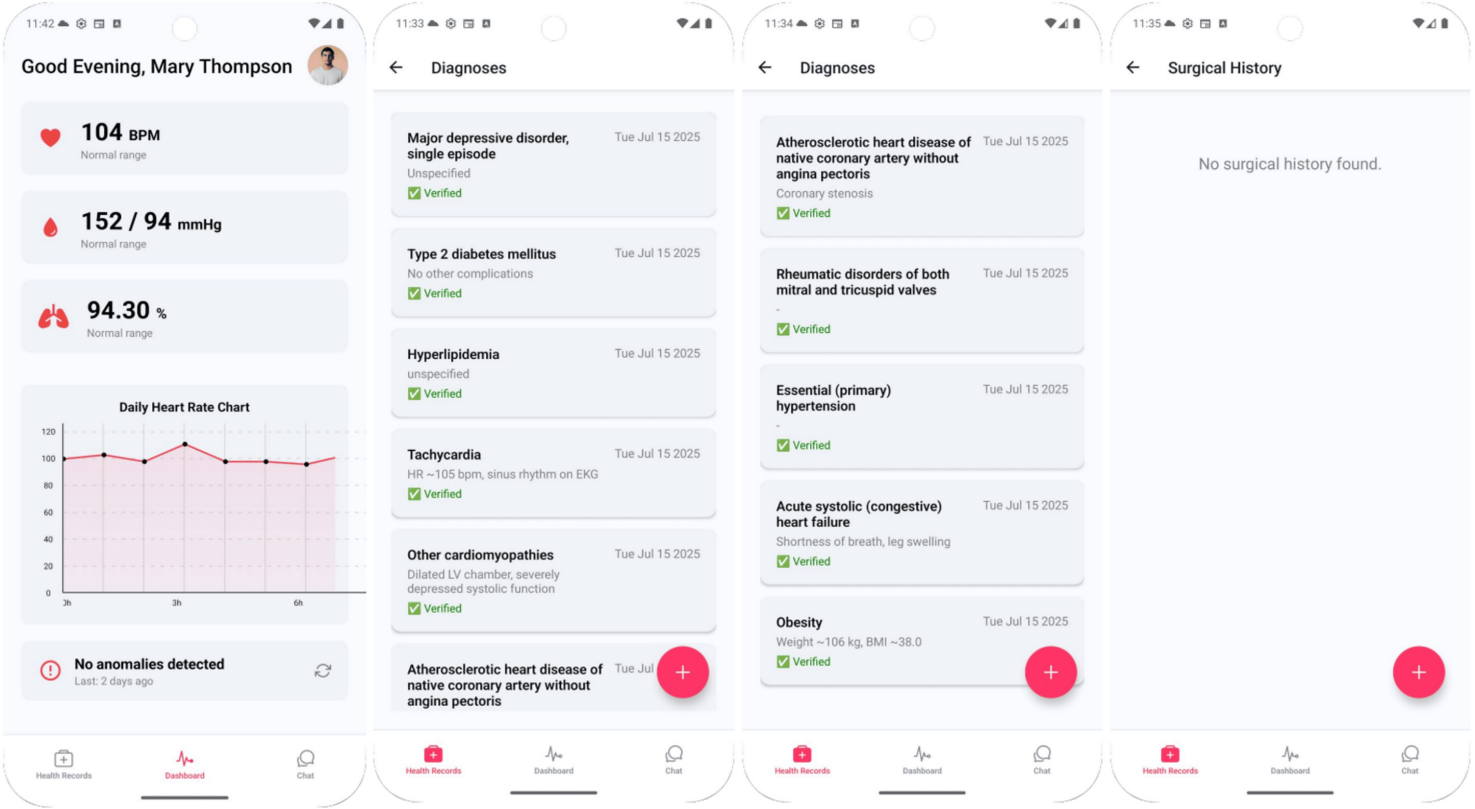
Dashboard overview with verified diagnoses and surgical procedures

Mary Thompson, a 54-year-old woman, 162 cm, and 106.4 kg, uses the application to understand her cardiometabolic conditions in context rather than as isolated labels. Her smartwatch-detected heart rate and blood pressure remain elevated, aligning with documented tachycardia and hypertension, and she augments passively collected device data with self-reports that her clinician later verifies against the EHR. Figure 2 presents Mary’s consolidated dashboard, showing her verified diagnoses, surgical history, and a longitudinal heart rate panel. This example illustrates how the system explains how hypertension and diabetes interact to worsen cardiovascular risk, linking explanations directly to her data. The dashboard emphasizes longitudinal trends, such as resting heart rate bands, so Mary can see how her daily life influences her physiology. Within the EHR view, each entry is time-stamped to reinforce temporality when interpreting the onset of the symptom.

**Figure 3:**
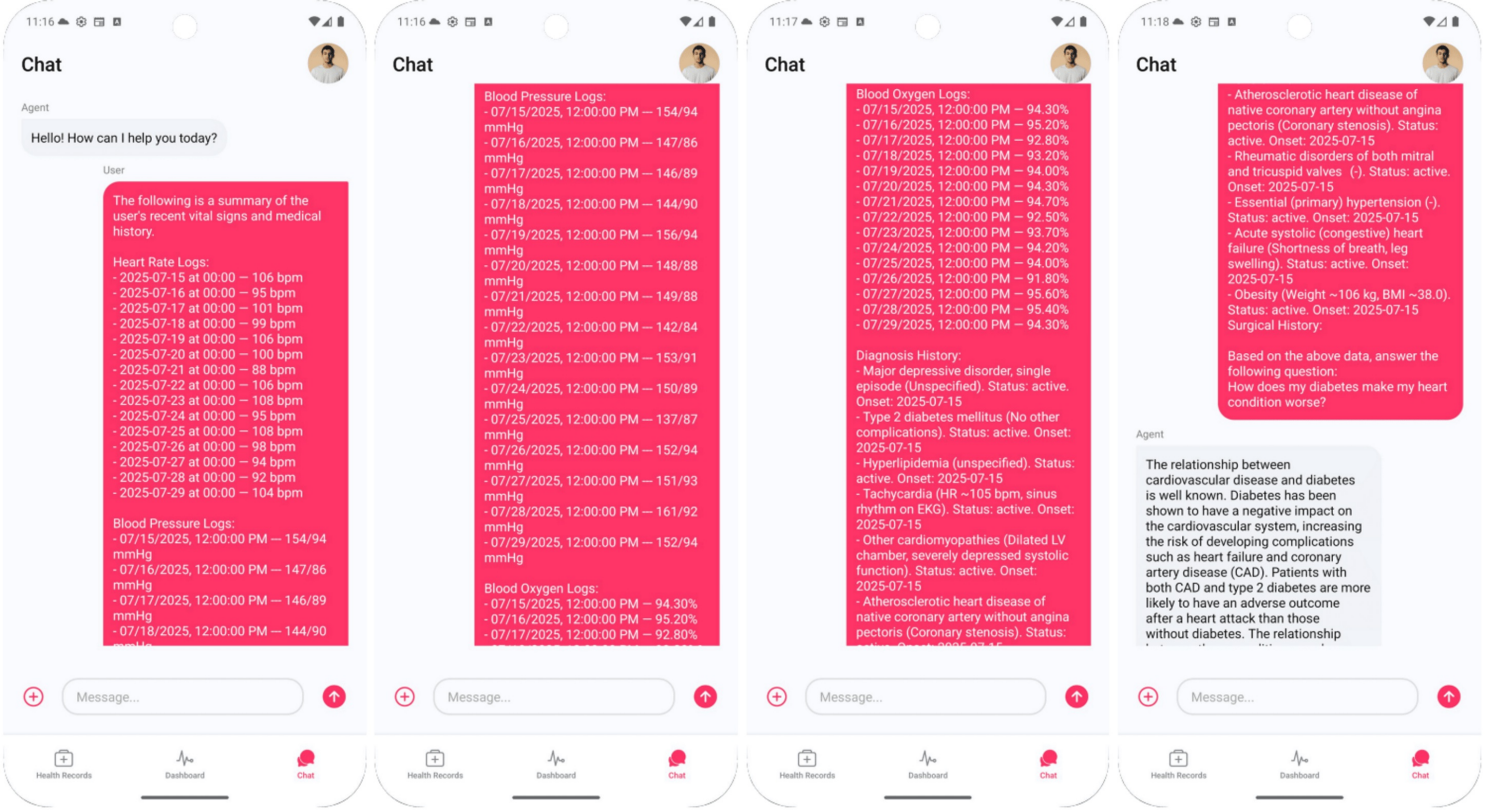
Prompt consisting of Mary’s data from Health Connect and electronic health record (EHR)

**Figure 4:**
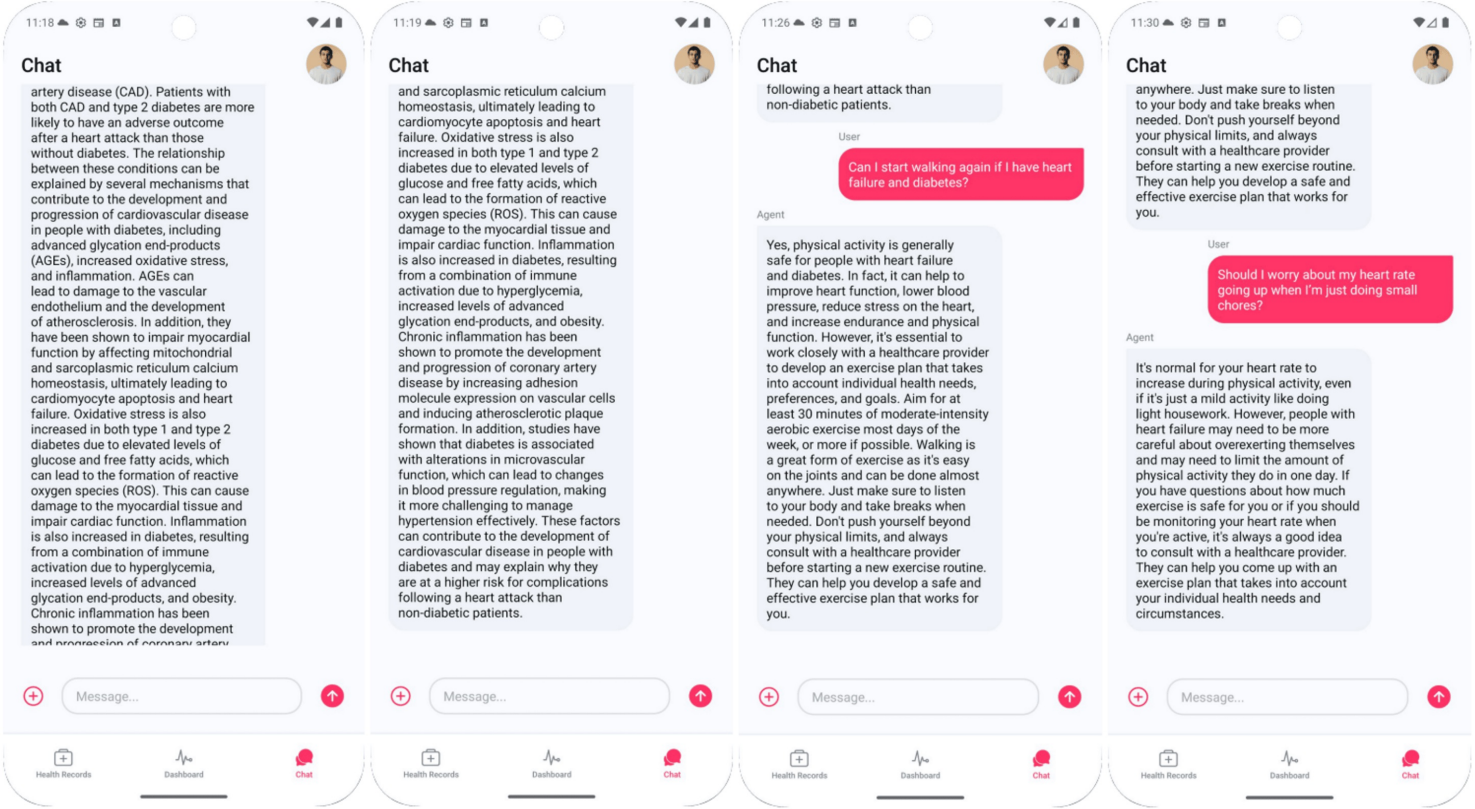
Example of AI-generated explanation: how diabetes exacerbates heart disease, grounded in patient’s own heart rate and problem list

Mary opens the chat and selects a date range. The application then compiles a structured prompt containing her vitals, active diagnoses list, and recent procedures before enabling free-text questions. Figure 3 illustrates the prompt, while Figure 4 presents the questions and responses by the LLM. The LLM generates responses by explicitly conditioning on both the patient’s history and current vital signs, while suppressing outdated or resolved items to reduce confounding. This targeted conditioning helps improve the specificity of answers.

When Mary asks, “How does my diabetes make my heart condition worse?”, the LLM explains that diabetes adversely affects cardiovascular risk and increases complications after myocardial infarction, then links the mechanisms to her data, as shown in Figure 4. It describes how chronic hyperglycemia can drive endothelial dysfunction and autonomic imbalance, plausibly contributing to blood pressure lability and perceived palpitations in Mary’s trend graphs.

Mary next asks whether light activity is safe given heart failure and diabetes, noting that heart rate rises with minimal exertion. The LLM normalizes a modest heart rate increase at initiation, recommends a conservative ramp-up under clinician oversight, and reiterates pre-specified escalation criteria symptoms that should trigger earlier contact. The plan proposes bout based walking with perceived exertion logging, instructs her to watch for dyspnea trends, and stores her responses as structured fields in Firebase so changes are computable rather than buried in free text. This way, she can get the instructions verified by a clinician, ensuring that educational content is coupled with safety actions rather than presented in isolation.

**Figure 5:**
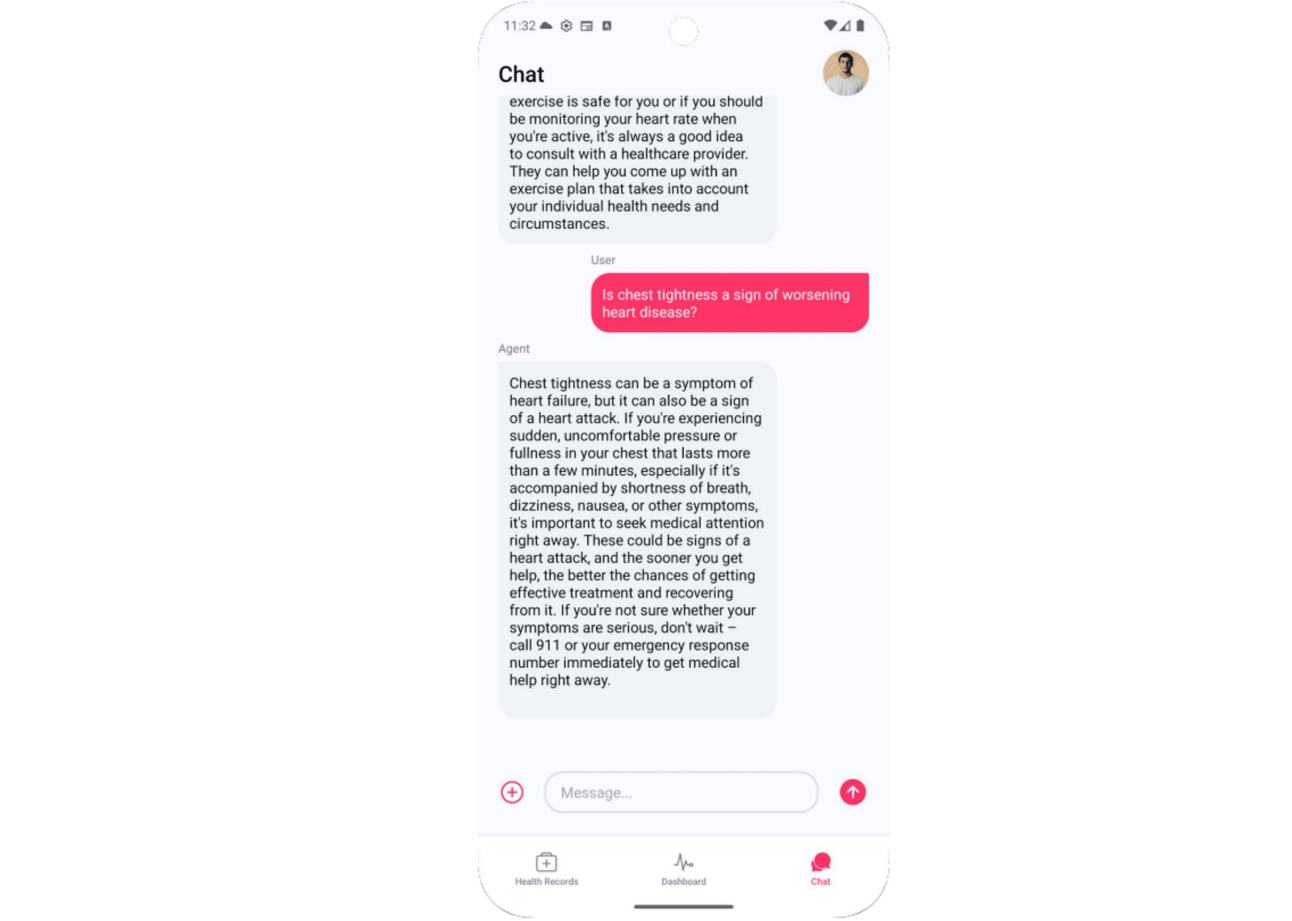
Large language model (LLM) response to potential chest tightening symptoms

Finally, as in Figure 5, Mary asks if chest tightness could signal worsening disease. The LLM clarifies that chest tightness may occur in heart failure but can also signal an acute coronary syndrome. Persistent pressure with shortness of breath, dizziness, or nausea warrants calling emergency services immediately. Outcome for education and engagement, Mary transitions from label-oriented comprehension to pattern-oriented self-management, linking daily measurements to risk while learning when to escalate promptly.

Use Case 2: Post-Surgical Recovery Monitoring

**Figure 6:**
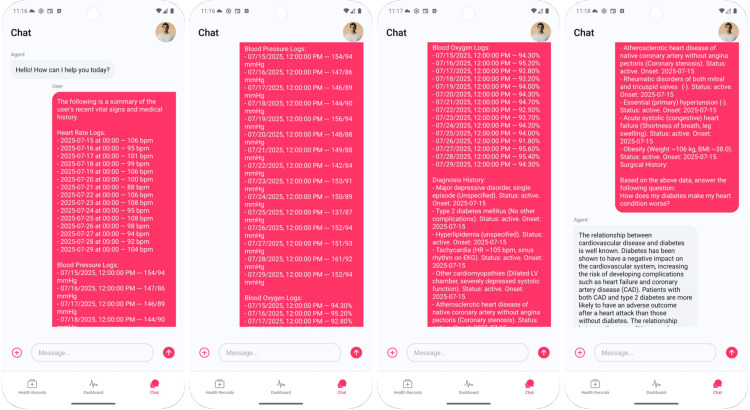
Dashboard and verified surgical history

John Doe (70 years, 177 cm, 82 kg) uses the app 10 days after angioplasty to make sense of early-recovery variability by combining smartwatch vitals with his EHR. The system ingests recent heart-rate, blood-pressure, and oxygen-saturation logs plus verified diagnoses/procedures, then injects a concise, structured summary directly into the chat and proposes a context-aware education prompt: “How does diabetes worsen my heart condition?” The LLM answers with a brief mechanism overview (Figure 6).

**Figure 7:**
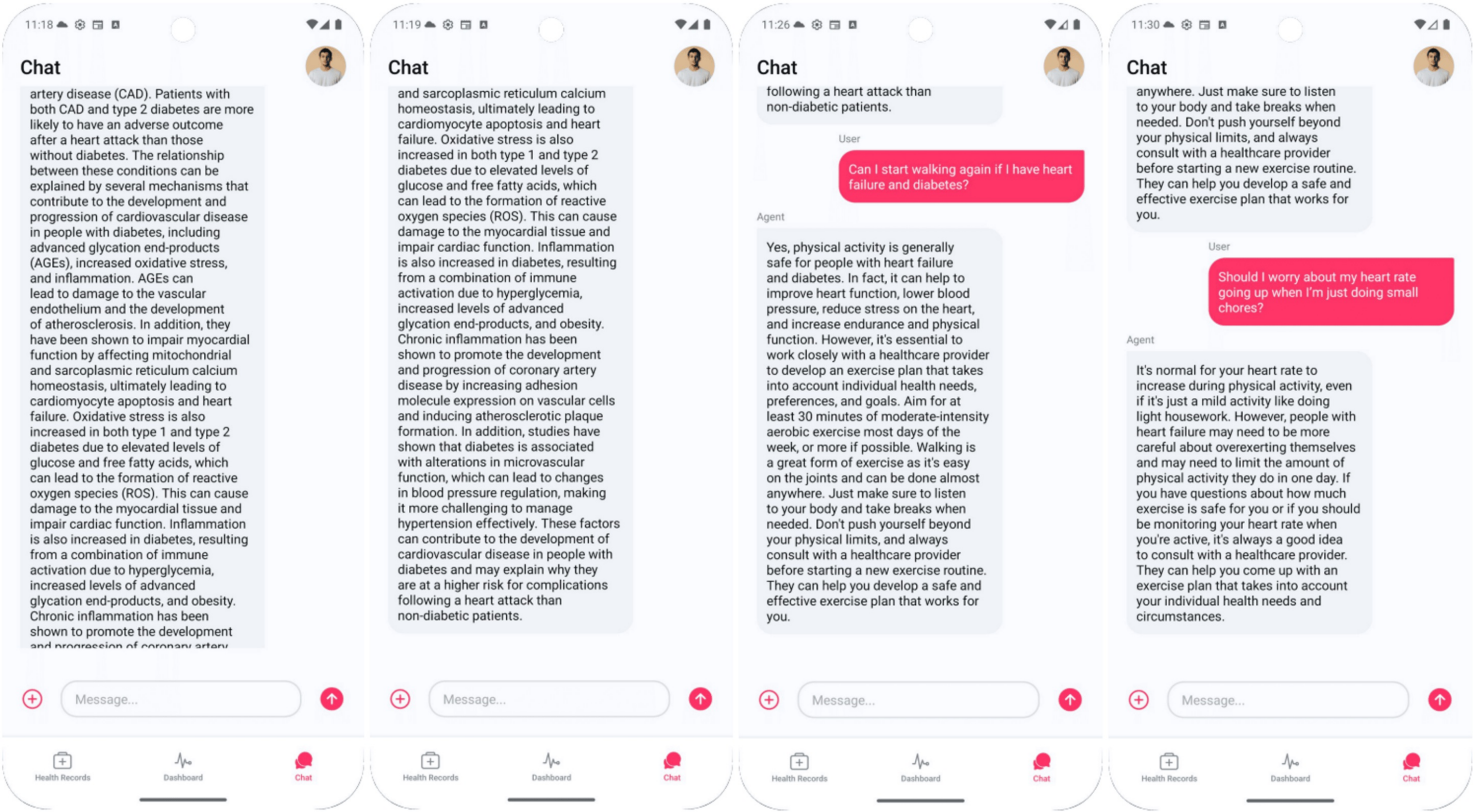
Verified diagnoses history

John then asks two practical questions: whether he can start walking with heart failure and diabetes, and whether a higher heart rate during light chores should worry him. The LLM provides conservative, plain-language guidance (written with template phrases intended to be broadly accessible; no formal readability scoring was performed in this study) emphasizing clinician-guided activity (e.g., moderate walking most days), listening to symptoms, taking breaks, and avoiding overexertion in heart failure, while normalizing transient heart-rate increases during routine tasks (Figure 7).

Together, Figures 6-7 show how the LLM, grounded in up-to-date vitals and EHR context, delivers targeted education and simple action guidance that helps John interpret expected fluctuations versus when to seek individualized advice from his care team. For clarity, Table 1 summarizes the two demonstrative personas, showing their conditions, data sources (simulated HR, BP, SpO₂; MIMIC-IV diagnoses), and the key explanation themes generated.

**Table 1:** Summary of demonstrative personas, data sources, and explanation themes

Persona (Use Case)	Conditions (from MIMIC-IV)	Data Sources Integrated	Explanation Themes Generated
Mary Thompson (Use Case 1)	Hypertension, type 2 diabetes, obesity	Simulated smartwatch heart rate and blood pressure; EHR problem list and procedures; self-reports verified against EHR	How diabetes exacerbates heart disease; effect of hypertension on cardiac risk; linking daily HR trends to lifestyle factors
Post-angioplasty patient (Use Case 2)	Coronary artery disease, post-stent procedure	Simulated smartwatch heart rate and SpO₂; synthetic blood pressure series; EHR surgical history	Understanding expected vs. concerning HR variability in recovery; guidance on safe daily activity; escalation when symptoms align with cardiac risk

## Discussion

Our integration of wearable data and LLM-driven analytics for cardiac monitoring demonstrates promise in enabling proactive, personalized cardiovascular care. As recent work affirms, coupling continuous multimodal sensor data with AI can shift cardiovascular diagnostics from episodic to continuous, patient-centered monitoring, potentially reducing delays in diagnosis and preventing hospitalizations and costs, but only if equity, usability, and integration challenges are addressed [16]. Consistent with that scope, our contribution is a workflow for patient-facing explanations and care navigation rather than a clinical evaluation.

A major innovation of our system lies in leveraging real-time wearable sensors alongside LLM interpretation to translate physiological anomalies into actionable clinician insights. This aligns with state-of-the-art systems such as "AI on the Pulse," which uses universal time-series anomaly detection models fused with LLM outputs to provide personalized, interpretable health alerts in real-world home settings [17]. Our architecture similarly harnesses interpretability to support clinician decision-making while mitigating black-box concerns.

Despite the considerable promise shown by AI-enabled wearable cardiovascular monitors, this study has specific limitations in addition to broader field-wide challenges. First, our evaluation relied on simulated smartwatch data and synthetic blood pressure series, meaning that no real patient outcomes were measured. Second, the use cases were limited to two scenario-based personas drawn from de-identified MIMIC-IV records, which restricts generalizability. Third, privacy-preserving deployment was not implemented in this prototype, and technical feasibility was demonstrated without usability testing. More generally, AI-wearable integration faces several limitations hindering translation into real-world clinical practice. Most existing studies remain small, short-term, and conducted under controlled conditions, constraining their applicability across diverse populations and settings, including remote [18]. From a technical standpoint, sensor accuracy is challenged by motion artifacts, poor contact, and environmental interference-issues that degrade algorithmic performance if not addressed by robust signal pre-processing [19].

Ethical and regulatory risks persist as well: patient privacy, algorithmic bias, data security, and regulatory ambiguity pose significant barriers to adoption [20]. Our work did not tackle the issue of privacy, as it was for demonstrative purposes. Our future work will include privacy considerations by employing techniques like using compact, on-device LLMs and/or federated learning. In addition, our planned work also includes running user studies with different and more advanced smartwatches (that can also capture blood pressure data) to validate the efficacy of our approach in the real world. Transforming wearable LLM systems from concept to clinical reality hinges on strengthening evidence, technical rigor, interoperability, and addressing ethical concerns such as privacy.

## Conclusions

Cardia-AI combines wearable signals with EHR context and translates that combined view through LLMs into concise, provenance-linked explanations bounded by safety guardrails. In two demonstrative use cases, the system illustrated how patients could connect symptoms to mechanisms, distinguish expected variability from warning patterns, and follow clearly stated escalation guidance without supplanting clinician judgment. Useful design choices include a select, compile, and ask workflow that yields auditable prompts and answers, retrieval-grounded generation with explicit data citations, and a default posture of education and escalation rather than diagnosis or prescription. The work has important limitations, including synthetic smartwatch data and de-identified EHRs, scenario-based validation, and no measurement of health outcomes or workflow impact. Accordingly, the conclusions should be read as feasibility observations only, not as evidence of clinical benefit or effectiveness. Future studies will be needed to evaluate subjective user experience and objective clinical outcomes to determine whether such a system helps patients in real-world settings. We also acknowledge that broader adoption depends on whether patients and clinicians view self-feedback loops as useful and how such systems can be integrated into current healthcare delivery models. In addition, future work will focus on privacy-preserving deployment through on-device models or federated learning, interoperability with production EHRs, human-factors evaluation of comprehension and trust, fairness analyses across devices and demographics, and prospective studies that quantify timeliness of escalation, clinician workload, and patient-reported outcomes. With such future studies, systems like Cardia-AI could progress from feasibility demonstrations toward rigorously evaluated tools, with the potential to make continuous cardiac monitoring clearer and more actionable.
